# Genome-wide analysis of HSP20 gene family and expression patterns under heat stress in cucumber (*Cucumis sativus* L.)

**DOI:** 10.3389/fpls.2022.968418

**Published:** 2022-08-12

**Authors:** Junjun Huang, Zhaoxin Hai, Ruoyi Wang, Yuanyuan Yu, Xin Chen, Weihong Liang, Huahua Wang

**Affiliations:** College of Life Science, Henan Normal University, Xinxiang, China

**Keywords:** cucumber, expression pattern, HSP20 gene family, heat stress, phylogenetic analysis

## Abstract

Cucumber is an important vegetable in China, and its yield and cultivation area are among the largest in the world. Excessive temperatures lead to high-temperature disorder in cucumber. Heat shock protein 20 (HSP20), an essential protein in the process of plant growth and development, is a universal protective protein with stress resistance. HSP20 plays crucial roles in plants under stress. In this study, we characterized the *HSP20* gene family in cucumber by studying chromosome location, gene duplication, phylogenetic relationships, gene structure, conserved motifs, protein-protein interaction (PPI) network, and *cis*-regulatory elements. A total of 30 *CsHSP20* genes were identified, distributed across 6 chromosomes, and classified into 11 distinct subgroups based on conserved motif composition, gene structure analyses, and phylogenetic relationships. According to the synteny analysis, cucumber had a closer relationship with *Arabidopsis* and soybean than with rice and maize. Collinearity analysis revealed that gene duplication, including tandem and segmental duplication, occurred as a result of positive selection and purifying selection. Promoter analysis showed that the putative promoters of *CsHSP20* genes contained growth, stress, and hormone *cis*-elements, which were combined with protein-protein interaction networks to reveal their potential function mechanism. We further analyzed the gene expression of *CsHSP20* genes under high stress and found that the majority of the *CsHSP20* genes were upregulated, suggesting that these genes played a positive role in the heat stress-mediated pathway at the seedling stage. These results provide comprehensive information on the *CsHSP20* gene family in cucumber and lay a solid foundation for elucidating the biological functions of CsHSP20. This study also provides valuable information on the regulation mechanism of the *CsHSP20* gene family in the high-temperature resistance of cucumber.

## Introduction

Heat shock proteins (HSPs) are ancient, highly conserved intracellular molecular chaperones that widely exist in almost all organisms (Dattilo et al., 2015). When organisms are subjected to environmental stresses such as salinity, high temperature, heavy metals, and drought, HSPs can improve the adaptation of organisms to stress by stabilizing the cell structure, transporting and folding auxiliary proteins and maintaining cell function (Tyedmers et al., 2010; Yadav et al., 2021). Under normal conditions, the content of HSPs accounts for only 5% of the total protein, but when subjected to external stress, the content increases rapidly to up to 15% (Zhang et al., 2015; Ul Haq et al., 2019).

Heat shock proteins can be divided into HSP100, HSP90, HSP70, HSP60, and sHSP (small HSP) families on the basis of amino acid sequence homology and molecular weight (Wang et al., 2004; Waters, 2013). sHSP is a kind of protein with a molecular weight of about 20 kDa, so it is also called HSP20 (Wang et al., 2004). All members of the HSP20 family contain conserved a-crystallin domain (ACD), which is encoded by about 90 amino acids, including a number of β-strand structures, a conversed 80–100 amino acid sequence, and two conserved regions: CRI (N-terminal consensus region I) and CRII (C-terminal consensus region II) (Bondino et al., 2012; Lin et al., 2019).

HSP20 is abundant in plants and the most produced protein under heat stress. It has been proven to be involved in plant response to biotic and abiotic stresses (Peffer et al., 2019; Wu et al., 2019; Ding et al., 2020; He et al., 2021). In *Arabidopsis*, *HSP17.4* was highly expressed after infection by *Pseudomonas syringae* (Sewelam et al., 2019). Overexpression of *AtHSP20s* could improve the tolerance to salt, oxidation, penetration, and high temperature by enhancing the activity of antioxidant enzymes (Mu et al., 2013; Zhang et al., 2013; Sun et al., 2016; Yang et al., 2020). In rice, the *OsHSP18.0* gene could strengthen the resistance to bacterial stripe disease by participating in the basic defense response (Ju et al., 2017). *OsHSP17*-overexpressing transgenic rice exhibited heat tolerance and UV resistance (Murakami et al., 2004). In tomato, overexpression of *SlHSP17.7* could enhance the tolerance to cold stress by inducing intracellular sucrose and reducing the accumulation of reactive oxygen species (Zhang et al., 2020). Cui et al. (2021) found the HSP20 gene family of African bermudagrass were related to abiotic stresses, and the *CtHSP20-9* and *CtHSP20-10* seemed to be positively selected in response to extreme temperatures. Similarly, when *JrsHSP17.3* was expressed, it conferred the tolerance to abnormal temperature and salt stresses by producing less H2O2 and MDA and accumulating more antioxidant enzymes and proline content (Zhai et al., 2016). Under high-temperature treatment, most *HSP20* genes were highly induced in pepper (Guo et al., 2015). Moreover, transgenic plants (*MsHSP16.9* and *TaHSP23.9*) grew vigorously and exerted a protective effect on *Arabidopsis* in high-temperature environments (Yang et al., 2017; Wang et al., 2020). Overall, these results have shown that HSP20 plays important positive roles in improving plant immunity and abiotic stress. In particular, HSP20 plays vital roles in mediating temperature stress tolerance.

According to the analysis of subcellular localization, sequence homology and phylogenetic relationship, the HSP20 family can be divided into at least 17 subgroups. Among them, 12 subgroups (CII-CXII) are localized to the nucleus/cytoplasm, and 5 subgroups (MI, MII, P, ER, and Px) are localized to the mitochondria, mitochondria, plastids (Ps), endoplasmic reticulum (ER), and peroxisomes (Pos) (Scharf et al., 2001; Ma et al., 2006; Siddique et al., 2008). However, some *HSP20* genes have not been classified into known subgroups. These unclassified *HSP20* genes may replicate newly generated genes with unique functions in plants and may represent *HSP20* subgroups in the early stage of evolution. Plants have more HSP20s than other eukaryotes, and the number varies in different species (Sarkar et al., 2009). With the completion of whole genome sequencing of numerous plants, comprehensive identification and analysis of the *HSP20* gene family in different species have been carried out. For example, 42, 48, 48, 41, 44, and 41 *HSP20* gene family members have been identified in the genomes of tomato (*Solanum lycopersicum*), potato (*Solanum tuberosum*), grape (*Vitis vinifera*), apple (*Malus domestica* Borkh), watermelon (*Citrullus lanatus*), and African bermudagrass (*Cynodon transvaalensis* Burtt-Davy), which were divided into 13, 12, 11, 10, 18, and 12 subgroups, respectively (Yu et al., 2016; He et al., 2018; Zhao et al., 2018; Ji et al., 2019; Yao et al., 2020; Cui et al., 2021).

Cucumber is a temperature-bias, widely cultivated crop with good economic benefits and plays an important role in the global vegetable supply. In recent years, the annual average temperature has increased annually due to global warming. High-temperature stress has become an important factor that inhibits the growth and development of cucumber (Ahuja et al., 2010; Zhu et al., 2017). Therefore, studying the resistance mechanism and resistance-associated genes of cucumber is crucial. The completion of high-quality genome assembly at the chromosome level of cucumber provides a powerful basis for studying the mechanism of the cucumber defense system at the genome-wide level (Li et al., 2019). This study identified *HSP20* genes in cucumber, systematically analyzed the characteristics of family members, including physical and chemical properties, domain, gene structure, phylogeny, and *cis*-regulatory elements (CREs) of the promoter, and investigated the expression of *HSP20* under high-temperature stress. The findings are expected to provide a reference for revealing the role of HSP family members in cucumber development regulation and stress response and serve as molecular resources for further cultivating new varieties that are resistant to high temperatures.

## Materials and methods

### Identification of HSP20 family members in cucumber

The latest cucumber genomic data (“Chinese Long” v3) were retrieved.^1^ The Hidden Markov Model (HMM) file of HSP20 was downloaded from Pfam,^2^ the local database was established, and the cucumber ACD domain with PF00011 (*e* value ≤ 1*e*^–20^) was searched. The probable CsHSP20 members were submitted to Pfam and SMART^3^ to further confirm the ACD domain. At the same time, the CsHSP20 protein sequences were submitted to https://web.expasy.org/protparam/ to determine the molecular weight. The CsHSP20s that lacked the ACD domain and had a molecular weight that exceeded 15∼42 KD were removed. The predicted CsHSP20s were named according to their molecular weight. Their physical and chemical properties were predicted using an online tool.^4^ Subcellular localization prediction was performed using ProtComp 9.0.^5^

### Phylogenetic analysis and classification of *CsHSP20* genes

The HSP20 protein sequences of rice and *Arabidopsis* were downloaded and combined with the identified CsHSP20 protein sequences for phylogenetic analysis. First, all of the protein sequences were aligned using MAFFT (version 7). Second, an un-rooted neighbor-joining (NJ) phylogenetic tree was constructed using MEGA-X with default parameters and a bootstrap test of 1000 times (Kumar et al., 2018). Lastly, the *CsHSP20* genes were classified into different subgroups on the basis of their subcellular localization predictions, classifications of HSP20s in other species, and topology of the phylogenetic tree.

### Conserved motif and gene structure analysis of *CsHSP20* genes

The gene structures of the *CsHSP20* genes were identified with an online tool^6^ (GSDS v2.0) (Hu et al., 2015). The conserved motifs of the CsHSP20s were identified using MEME Suite (version 5.4.1^7^). The maximum motif number was 10, and the default parameters were used for other parameters.

### Chromosomal locations, gene duplications, and synteny analysis

All of the proposed *CsHSP20* genes were assigned to cucumber chromosomes on the basis of Cucumber (Chinese Long) v3 Genome and its annotation file. Gene duplication of *CsHSP20* and collinearity analysis was performed with TBtools (Chen et al., 2020a). DnaSP_v61203 was used to calculate *Ka* and *Ks*. The formula “*T* = *Ks*/(2λ × 10^–6^)” (λ = 6.5 × 10^–9^) was used to calculate the occurrence time of each duplicated gene pair (Wang et al., 2015).

### Analysis of *cis*-regulatory elements in *CsHsp20* genes’ putative promoter regions

In order to study the potential regulatory mechanism of *CsHSP20* genes in abiotic stress response, 2 kb upstream sequences were downloaded from Cucurbit Genomic Database (see text footnote 1) and then submitted to PlantCARE^8^ to identify the CREs (Lescot et al., 2002). At the same time, the heat-shock elements (HSEs) were searched using the motif-based sequence analysis tool MEME and *via* manual inspection.

### Protein-protein interaction network

The sequences of CsHSP20 protein were submitted to STRING^9^ (v11.5) to predict the relationships between CsHSP20 proteins and other related proteins (Szklarczyk et al., 2019). The organism was set to *Arabidopsis*, and the advanced settings remained in the default mode. The PPI networks were constructed and visualized with Cytoscape v3.9.0 (Shannon et al., 2003).

### Plant materials and heat stress treatment

North China cucumber cultivar Jinchun No. 4 seeds were soaked in an incubator under room temperature for 2 days. The germinated seeds were then grown on a nursery tray under standard greenhouse conditions (light/dark cycle: 16/8 h at 28°C) for 2 weeks. The uniform seedlings were transferred to a growth chamber maintained at 45°C for heat stress. The first true leaf was collected at 0, 3, 6, and 12 h and used for quantitative real-time PCR (qRT-PCR) analysis to detect the expression of *CSHSP20* genes with *CsActin* as internal control. The cycling steps were 95°C for 5 min, followed by 40 cycles of 10 s at 95°C and 30 s at 60°C, and at the end, one cycle of 10 s at 95°C, 1 min at 65°C, and 15 s at 97°C. Each treatment was carried out in three independent replicates, and samples of five plants were collected in each replicate. The primers were listed in Supplementary Table 1.

## Results

### Genome-wide identification of HSP20 genes in cucumber

On the basis of the published cucumber (*Cucumis sativus* L. var. *sativus* cv. 9930) genome (Li et al., 2019), a total of 30 HSP20 genes were identified and confirmed with the ACD domain by using SMART, CDD, and Pfam (Letunic and Bork, 2018). Sequences with a molecular weight of more than 15–42 kDa were removed. The names of the CsHSP20s were determined according to their molecular weight (MW) (Table 1). Detailed information on the characteristics of the CsHSP20s is presented in Table 1. Overall, the identified cucumber CsHSP20 genes encoded protein with a size ranging within 130–356 amino acid residues. The theoretical isoelectric point (pI) and MW of the CsHSP20 proteins varied from 4.51 (CsHSP16.4) to 9.55 (CsHSP23.4) and from 15.03 kDa (CsHSP15.0) to 39.89 kDa (CsHSP39.8), respectively.

**TABLE 1:** The basic information of CsHSP20 proteins identified in cucumber.

Named	Gene ID	Size (AA)	Strand	Location	Isoelectric point	Molecular weight (kDA)	Subcellular localization
CsHSP18.8	CsaV3_1G005060	168	+	3382876–3383465	9.46	18.82	Extracellular
CsHSP16.4	CsaV3_1G035810	148	−	21892088–21893838	4.51	16.49	Nuclear
CsHSP18.2	CsaV3_1G035820	160	−	21895476–21896585	6.13	18.21	Cytoplasmic
CsHSP15.4	CsaV3_1G035830	135	+	21898380–21898862	6.21	15.41	Cytoplasmic
CsHSP39.8	CsaV3_1G041750	356	−	26652841–26654260	9.21	39.89	Extracellular
CsHSP39.5	CsaV3_1G041760	355	−	26658938–26660854	9.45	39.57	Extracellular
CsHSP23.8A	CsaV3_3G002770	212	+	2251829–2254144	5.43	23.80	Chloroplast
CsHSP23.8B	CsaV3_3G002780	212	+	2255195–2257043	5.26	23.83	Chloroplast
CsHSP22.9	CsaV3_3G005420	200	+	4551319–4554535	4.86	22.98	Cytoplasmic
CsHSP29.9	CsaV3_3G006720	265	−	5920734–5922774	7.74	29.99	Endoplasmic reticulum
CsHSP16.3	CsaV3_3G027480	144	+	23792753–23793946	6.84	16.31	Cytoplasmic
CsHSP23.6	CsaV3_3G034390	200	+	29181040–29182090	8.34	23.67	Nuclear
CsHSP20.3	CsaV3_3G034400	172	+	29183236–29183754	9.01	20.33	Extracellular
CsHSP23.9	CsaV3_4G001560	210	+	880036–880668	5.49	23.99	Mitochondrial
CsHSP15.0	CsaV3_4G017440	130	−	10885738–10886969	8.6	15.03	Extracellular
CsHSP15.9	CsaV3_4G025700	143	−	15114379–15114810	6.76	15.91	Cytoplasmic
CsHSP36.1	CsaV3_4G035900	323	−	25245251–25248160	7.69	36.13	Cytoplasmic
CsHSP25.5	CsaV3_5G000040	229	+	35597–38762	9.12	25.51	Chloroplast
CsHSP25.0	CsaV3_5G008680	222	−	5339660–5344162	9.08	25.04	Cytoplasmic
CsHSP17.3	CsaV3_5G008690	152	+	5349881–5350786	9.27	17.38	Cytoplasmic
CsHSP21.3	CsaV3_5G008700	189	−	5360693–5363946	6.02	21.38	Cytoplasmic
CsHSP23.4	CsaV3_5G008750	202	−	5433169–5433777	9.55	23.42	Cytoplasmic
CsHSP18.1	CsaV3_5G008760	159	−	5435242–5437328	6.21	18.11	Nuclear
CsHSP22.2B	CsaV3_5G008770	191	+	5435387–5435962	6.47	22.29	Nuclear
CsHSP17.8	CsaV3_5G008780	156	+	5443251–5444088	5.97	17.83	Nuclear
CsHSP21.1	CsaV3_5G030610	183	+	25114220–25116088	4.93	21.11	Chloroplast
CsHSP38.0	CsaV3_5G035060	352	−	27756422–27758582	4.94	38.00	Extracellular
CsHSP17.9	CsaV3_6G001540	154	+	1008796–1012576	6.77	17.95	Cytoplasmic
CsHSP27.0	CsaV3_6G037900	246	+	21463616–21466526	7.6	27.06	Extracellular
CsHSP22.2A	CsaV3_7G008210	197	−	5140602–5141331	6.87	22.22	Cytoplasmic

### Chromosomal distribution and synteny analysis of CsHSP20s

**FIGURE 1:**
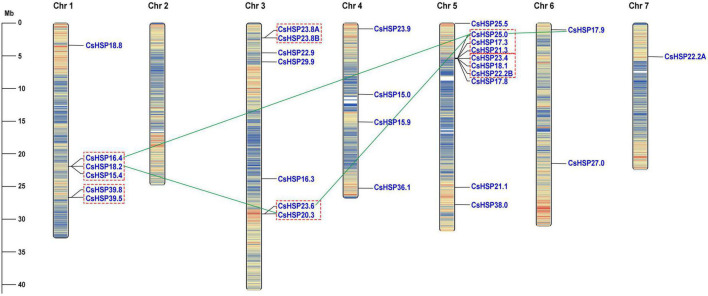
Chromosomal locations of *CsHSP20* genes. Tandem duplicated genes are indicated by red dashed box and segmental duplicated genes are connected by green line. Chromosome numbers are shown at the top of each bar. The scale presented on the left indicates chromosome sizes in mega bases (Mb).

The *CsHSP20* genes were found to be unevenly distributed across seven cucumber chromosomes (Figure 1). Chromosome 1 contained 6 *CsHSP20s*, chromosome 3 contained 7 *CsHSP20s*, and chromosome 5 contained 10 *CsHSP20s*. The four other chromosomes contained only four, two, one, and even no *CsHSP20* genes (Chr 2). Most of the *CsHSP20* genes were located at both ends of the chromosomes. This distribution was similar to that of gene density. The gene density at both ends of the chromosomes was large, but the density in the middle was small (Figure 1).

We analyzed the locations of CsHSP20 duplicates in the cucumber genome. Overall, 16 (53%) *CsHSP20* genes exhibited tandem or segmental duplication. As shown in Figure 1, we identified four pairs of segmental duplicates on chromosomes 1, 3, 5, and 6: CsHSP18.2/CsHSP20.3, CsHSP16.4/CsHSP25.0, CsHSP23.6/CsHSP25.0, and CsHSP25.0/CsHSP17.9. In addition, nine gene pairs (CsHSP16. 4/CsHSP18.2, CsHSP18.2/CsHSP15.4, CsHSP39.8/CsHSP39.5, CsHSP23.8A/CsHSP23.8B, CsHSP23.6/CsHSP20.3, CsHSP25.0 /CsHSP17.3, CsHSP17.3/CsHSP21.3, CsHSP23.4/CsHSP18.1, CsHSP18.1/CsHSP22.2B) seemed to occur through tandem duplication. We also determined the duplicate divergence times by calculating the *Ka*/*Ks* ratios. Tandem duplication event occurred in the past 50 MYA, whereas segmental duplicates occurred earlier, especially CsHSP23.6/CsHSP25.0 and CsHSP25.0/CsHSP17.9 from more ancient duplication events. All of the CsHSP20s paralogs, except for CsHSP23.6/CsHSP20.3, experienced purifying selection (*Ka*/*Ks* < 1) (Table 2).

**TABLE 2:** *Ka/Ks* calculation and divergent time of the duplicated cucumber *HSP20* gene pairs.

Duplicated gene pairs	Duplicated type	*Ka*	*Ks*	*Ka/Ks*	Purify selection	Time * (MYA)
CsHSP16.4/CsHSP18.2	Tandem	0.4865	2.9041	0.1675	Yes	221.35
CsHSP18.2/CsHSP15.4	Tandem	0.0586	0.2807	0.2088	Yes	21.39
CsHSP39.8/CsHSP39.5	Tandem	0.1454	0.2153	0.6753	Yes	16.41
CsHSP23.8A/CsHSP23.8B	Tandem	0.0472	0.2244	0.2103	Yes	17.1
CsHSP23.6/CsHSP20.3	Tandem	0.2937	0.2767	1.0614	Yes	21.09
CsHSP25.0/CsHSP17.3	Tandem	0.0841	0.6262	0.1343	Yes	47.73
CsHSP17.3/CsHSP21.3	Tandem	0.0699	0.4006	0.1745	Yes	30.53
CsHSP23.4/CsHSP18.1	Tandem	0.0681	0.4096	0.1663	Yes	31.22
CsHSP18.1/CsHSP22.2B	Tandem	0.2611	0.9184	0.2843	Yes	70
CsHSP18.2/CsHSP20.3	Segmental	0.6771	1.5501	0.4368	Yes	118.15
CsHSP16.4/CsHSP25.0	Segmental	0.5231	2.3015	0.2273	Yes	175.42
CsHSP23.6/CsHSP25.0	Segmental	0.6852	4.9858	0.1374	Yes	380.02
CsHSP25.0/CsHSP17.9	Segmental	0.1663	4.2341	0.0393	Yes	322.72

**FIGURE 2:**
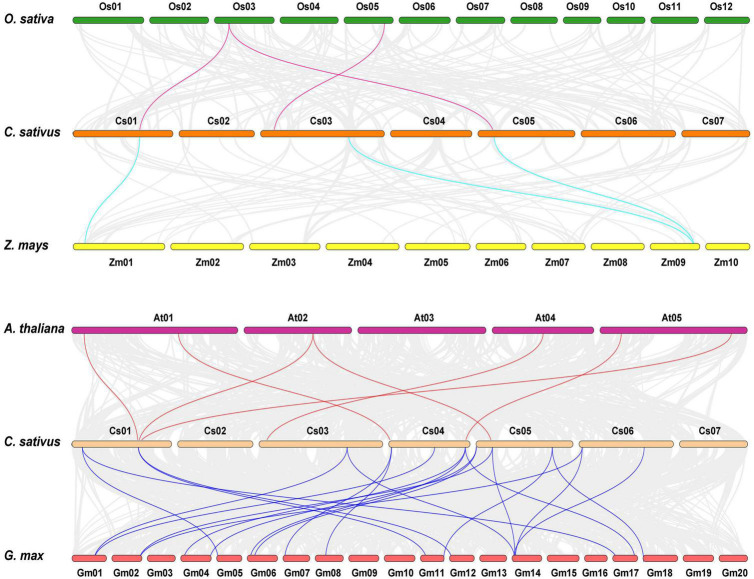
Synteny analysis of HSP20 gene family between *Cucumis sativus*, *Oryza sativa*, *Zea mays*, *Glycine max*, and *Arabidopsis thaliana*. The gray lines indicated collinear blocks within cucumber genome and other plant genomes, while the colored lines showed the syntenic HSP20 gene pairs.

Comparative syntenic maps of cucumber related to four representative species, namely, two dicots (*Arabidopsis thaliana* and *Glycine max*) and two monocots (*Oryza sativa* and *Zea mays*), were created to explore the phylogenetic mechanisms of CsHSP20 family (Figure 2). The results revealed that 3, 3, 7, and 21 HSP20 collinear gene pairs were identified in cucumber and rice, cucumber, and maize, cucumber and *Arabidopsis*, and cucumber and soybean, respectively (Figure 2 and Supplementary Table 2). Half of the *CsHSP20s* had syntenic genes in at least one of the four species, and most of them had syntenic genes in only one species. The *Ka*/*Ks* values of all of the syntenic gene pairs were less than 1, indicating that they might have experienced purifying selection. Moreover, only one *CsHSP25.0* exhibited orthologous genes across in all of the four species (Supplementary Table 2). The remaining half of the *CsHSP20s* had no syntenic relationships with the four species, suggesting that they are conservative members of the CsHSP20 gene family.

### Phylogenetic analysis of the *CsHSP20* gene family in cucumber

**FIGURE 3:**
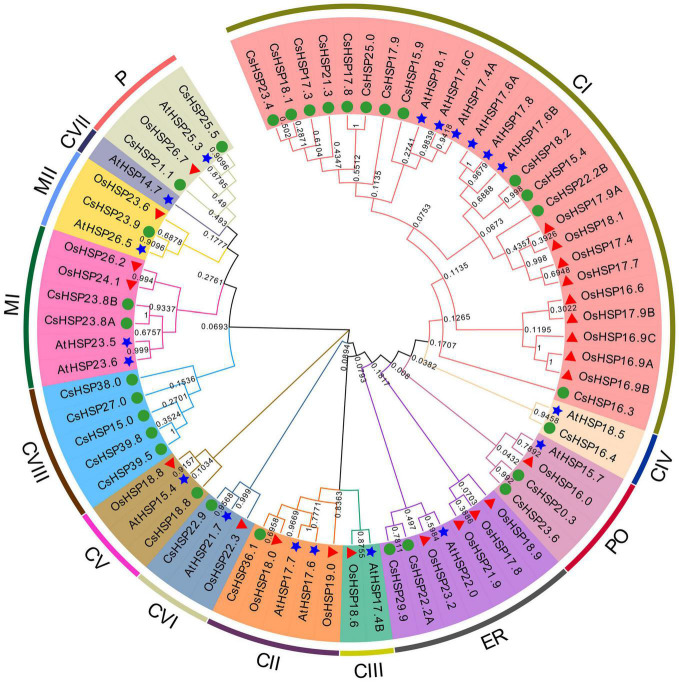
Phylogenetic tree of the total HSP20 proteins from cucumber, *Arabidopsis* (19 AtHSP20), and rice (23 OsHSP20). The NJ phylogenetic tree was constructed by MEGA-X with default parameters and bootstrap test of 1000 times. The arcs marked with distinct color represent different subgroups of HSP20 proteins. The green circle, red triangle, and blue star represent HSP20 from cucumber, rice, and *Arabidopsis*, respectively.

To thoroughly understand the evolutionary relationship of the HSP20 gene family, an unrooted neighbor-joining phylogenetic tree was established using MEGA X on the basis of the alignment of the amino acid sequences of HSP20 from cucumber, *Arabidopsis* (19 sequences), and rice (23 sequences) (Figure 3). The CsHSP20 proteins were divided into 11 subgroups, including 12 CI, 5 CVIII, 2 MI, 2 ER, 2 P, 2 PO and one each of CII, CV, CVI, CIV, MII based on previously reported results and phylogeny analysis (Figure 3; Ji et al., 2019; Yao et al., 2020). The clustering of the subfamilies in cucumber was basically consistent with the subcellular localization predicted by the online tool ProtComp 9.0 (Table 1 and Figure 3). About half of the CsHSP20s were localized in the cytoplasm, which meant that they mainly played roles in the cytosol. CsHSP16.4 belonged to the CIV subgroup that exists only in dicot plants. The result is consistent with previous findings (Siddique et al., 2008; Zhao et al., 2018; Yao et al., 2020).

### Gene structure and conserved motifs analysis

**FIGURE 4:**
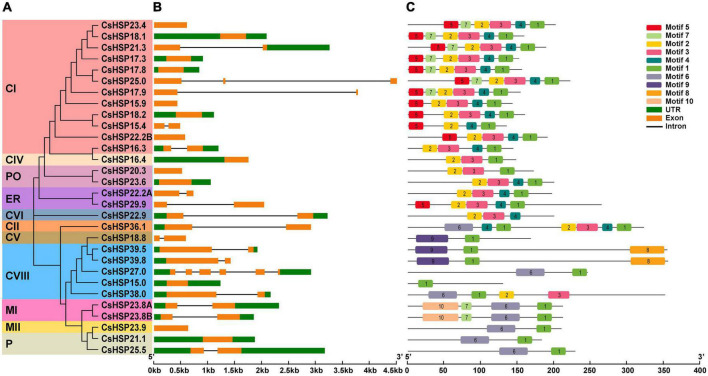
Gene structure and conserved protein motifs of HSP20 in cucumber. **(A)** The NJ phylogenetic tree of CsHSP20. The different colors represent the different subgroups. **(B)** Gene structure of *CsHSP20* genes. The untranslated regions, introns, and exons are indicated by green boxes, golden boxes, and solid gray lines, respectively. The scale at the bottom is in kp. **(C)** Conserved motifs in CsHSP20 proteins. Boxes of different colors represent different motifs. Details of each motif are shown in Supplementary Table 2.

GSDS v2.0 (Hu et al., 2015) analysis of the intron/exon structure of the *CsHSP20s* showed that almost all members of the *CsHSP20* genes contained only zero or one intron (13 with no intron, 15 with oner intron, one with two introns, and one with five introns) (Figure 4B). The different *CsHSP20* genes in each subgroup exhibited similar exon-intron structures, indicating their high conservatism and close evolutionary relationships (Figures 4A,B).

In order to better understand the CsHSP20s, the 30 sequences of CsHSP20s were imported to MEME software to predict the conserved motifs. The results showed that the length of motifs was between 15 and 50 aa (Supplementary Table 3) and the CsHSP20s in the same subgroup had similar motif compositions and order of arrangement (Figure 4C). Each CtHSP20 had motif 1. Motif 5 was found to be specific to subgroups CI and ER. Motif 7 was unique to subgroups CI and MI. Motifs 8 and 10 were found to be unique to subgroups CVIII and MI, respectively. Motif 9 was specific to subgroups CV and CVIII. Overall, the CsHSP20 proteins in the same subgroup had a similar domain composition and arrangement, which implies that they might have similar functions. Individual differences mean functional differences.

### Protein–protein interaction network

**FIGURE 5:**
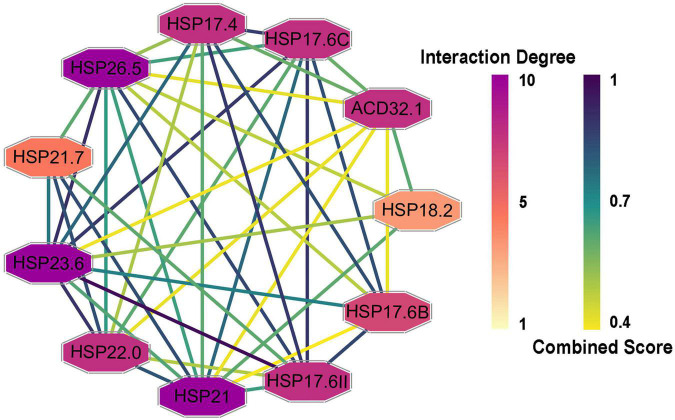
Protein-protein interaction (PPI) networks of CsHSP20s and their related proteins. The protein node color indicated the interaction degrees linked with each node, and the edge color represented the combined score, the darker the color, the greater the value.

To determine the interactions among the CsHSP20s and related proteins, a PPI network was constructed and visualized using the STRING database and Cytoscape software, respectively (Shannon et al., 2003; Szklarczyk et al., 2019). In total, 26 proteins had *Arabidopsis* orthologs with identities from 33.7 to 78.1% (Supplementary Table 4). As shown in Figure 5, the PPI network consisted of 11 nodes and 43 edges, suggesting that the proteins were highly linked with other proteins and participated in some biological processes. For example, the interaction degree of HSP26.5, HSP21, and HSP23.6 was 10, and that of HSP18.2 was 4.

### Promoter analysis of *CsHSP20s*

**FIGURE 6:**
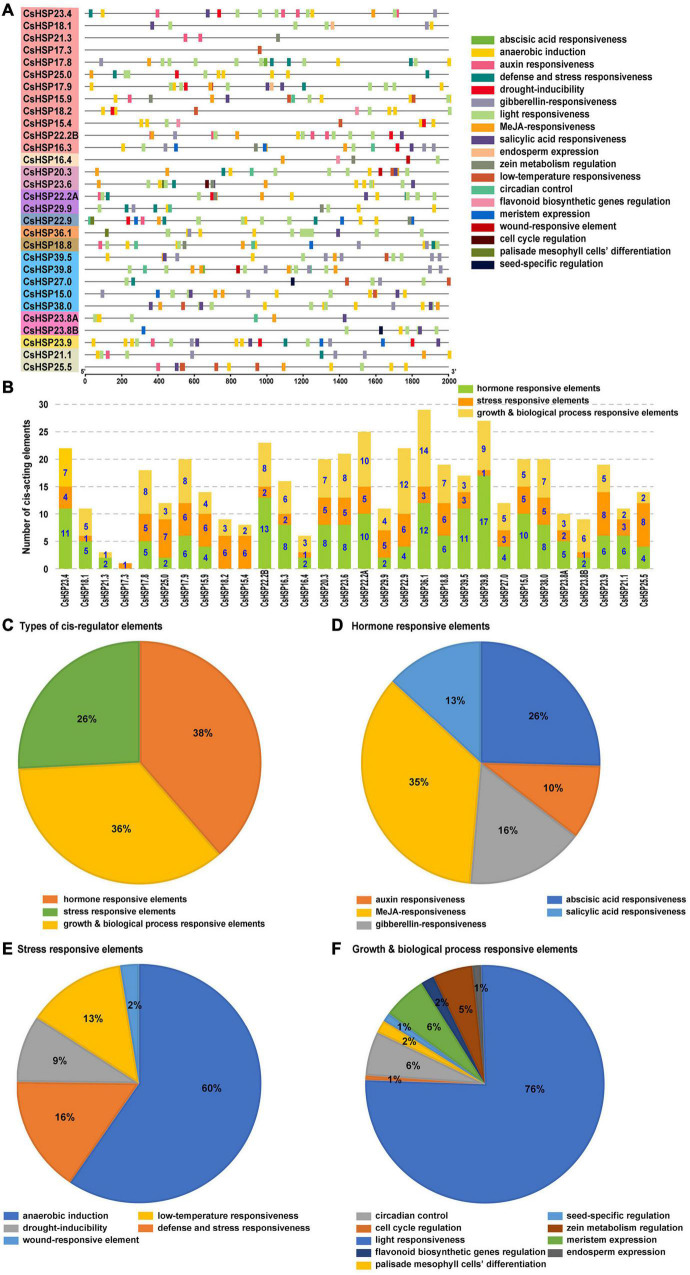
Statistical summary and distribution of *cis*-elements. **(A)** *Cis*-element distributions in the putative promoters of *CsHSP20s*. **(B)** The number of three kinds of *cis*-elements in the promoters of each *CsHSP20* gene. **(C)** The relative proportions of three kinds of *cis*-elements. **(D–F)** The detailed percentages of each type of *cis*-element.

For the *CsHSP20* genes, a total of 469 CREs were predicted and classified into three main types: hormone-responsive (auxin, MeJA, GA, ABA, and SA), stress-responsive (anaerobic induction, defense and stress, low temperature, wound, and drought), and growth and biological process-responsive elements (circadian control, cell cycle, and light, etc.; Figure 6 and Supplementary Table 5). The number of different CREs in each *CsHSP20* gene’s promoter is shown in Figures 6A,B. Basically, each *CsHSP20* gene contained multiple CREs aside from *CsHSP17.3* and *CsHSP21.3*. The proportion of three types of CREs was almost the same (Figure 6C). As shown in Figure 6D, the *CsHSP20s* gene expression was mainly regulated by MeJA, followed by ABA, GA, SA, and IAA hormone. Notably, the CRE essential for anaerobic induction and the CRE involved in light responsiveness accounted for 60 and 76%, respectively (Figures 6E,F). HSP genes can usually be induced to express for the heat shock transcription factors (HSFs) binding HSEs. Eukaryotes have three types of HSEs: perfect (P-type, nGAAnnTTCnnGAAn or TTCnnGAAnnTTC), gap [G-type, nTTCnnGAAn (5 bp) nGAAn], and step [S-type, nTTCn (5 bp) nTTCn (5 bp) nTTCn] (Mittal et al., 2011). From the manual inspection and the motif-based sequence analysis tool MEME, we found that only two HSEs, namely, P- and S-type HSEs, existed in CsHSP18.2 and CsHSP22.2B, respectively. Among all presumptive CREs, the different elements related to developmental processes accounted for the smallest proportion, that is, only 3.62% (Figure 6F and Supplementary Table 5). These results indicate that the ubiquitous CREs in *CsHSP20s*’ promoters might function in gene expression regulation related to multiple stresses and in growth and development.

### Expression profiling of *CsHSP20* genes under heat stress treatment

**FIGURE 7:**
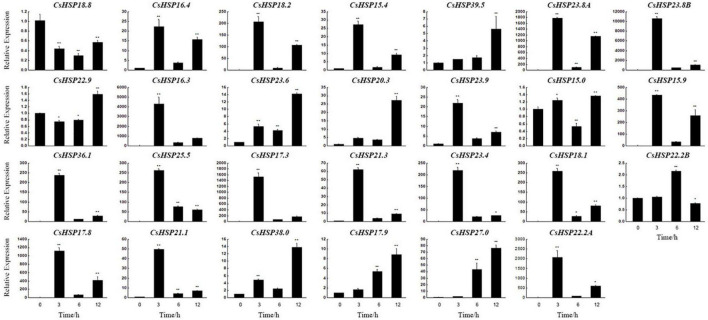
The relative expression levels of cucumber *HSP20* genes from qRT-PCR under heat stresses. The first true leaf of two-seedling exposed to 45°C for 0, 3, 6, and 12 h was used for analysis. All data shown are means SD of three biological replicates. The asterisks indicate the significant level (*P < 0.05, **P < 0.01) based on a Duncan’s multiple range test.

To explore the potential functions of *CsHSP20s* in response to heat stress, we performed a *CsHSP20s* expression analysis after heat treatment by qRT-PCR. We observed that except for *CsHSP29.9* and *CsHSP25.0*, all other genes were sensitive to heat stress and their relative expression level fluctuated during the 12 h treatments (Figure 7). The expression levels of most *CsHSP20* genes considerably increased and reached at a high level after short-term heat stress (45°C for 3 h); the expression levels were more than 20-fold, or even 10,000-fold higher than those under normal condition. The expression of minority *CsHSP20* genes increased gradually. *CsHSP18.8* was downregulated under heat stress, but the expression level of *CsHSP15.0* changed only slightly. The expression level of *CsHSP39.8* was not detected at 3 and 6 h, but it reached the highest value at 12 h. Overall, the vast majority of the *CsHSP20* genes responded to the heat stress treatment.

## Discussion

HSP20, a class of molecular chaperones, is ubiquitous and the most abundant HSP family involved in abiotic stress in plants (Waters, 2013; Zhao et al., 2018). *HSP20* genes have been identified in dicot and monocot plants, such as potato (Zhao et al., 2018), apple (Yao et al., 2020), tomato (Yu et al., 2016), grape (Ji et al., 2019), watermelon (He et al., 2018), African bermudagrass (Cui et al., 2021), and red algae (Gao et al., 2022). However, the knowledge on *HSP20* genes in cucumber, an important vegetable crop, is limited. In this study, we identified 30 CsHSP20 family genes, more than in *Arabidopsis* (9), but fewer than in most other species, such as apple (41) (Yao et al., 2020), African bermudagrass (41) (Cui et al., 2021), and grape (48) (Ji et al., 2019). The difference in the number of *HSP20* genes is probably due to the difference in genome size and gene replication in the process of plant evolution. The genome size of *Arabidopsis* is relatively small, with only 6 *HSP20* genes (Zhao et al., 2018).

Segmental duplication, tandem duplication, and whole-genome duplication events, especially the former two events, mainly contribute to the expansion of the gene family in plants (Cannon et al., 2004; Maere et al., 2005). We identified 13 gene pairs, including 4 segmental duplication and 9 tandem duplication gene pairs, constituted by 16 *CsHSP20s* (16/30, 53.3%) that were located on Chr 1, Chr 3, Chr 5, and Chr 6 (Figure 1 and Table 2). This result meant that Chr 1, Chr 3, and Chr 5 had the most duplication events, which could partly explain the larger numbers of *HSP20* genes on Chr 1, Chr 3, and Chr 5. Meanwhile, the remaining chromosomes had no or only a few *HSP20* genes. The result also meant that tandem duplication contributed more to the expansion of CsHSP20 families than segmental duplication.

It had been reported the CCT (cucurbit-common tetraploidization, 90∼102 MYA) event plays a vital role in the establishment of all Cucurbitaceae plants (Wang et al., 2018). However, we found that segmental duplication occurred before the core-eudicot-common hexaploidization event (ECH, 115∼130 MYA), and tandem duplication occurred after the CCT event, indicating that the two events had no effect on the expansion of the *CsHSP20* gene family (Table 2). This result may explain why the CsHSP20 gene family is small. It also explains why many other cucumber gene families are quite small (Chen et al., 2020b; Cheng et al., 2020; Zhang et al., 2021). Half of the *CsHSP20* genes were conservative and did not have collinearity with the *HSP20* genes in the four tested species. However, the other half of the *CsHSP20* genes showed collinearity with the 2, 2, 5, and 18 *HSP20* genes in rice, maize, *Arabidopsis*, and soybean, respectively, which were orthologous genes (Figure 2 and Supplementary Table 2). These results suggest that the expansion of the *HSP20* gene family in each species was carried out in a specific way. The orthologous gene pairs provided effective information on the evolutionary relationships among species (Yu et al., 2014).

According to the phylogenetic relationships with *Arabidopsis* and rice, gene structures, and amino acid sequences, the 30 CsHSP20 proteins were similar to typical HSP20 family proteins in other species with classifications into 11 subgroups (Figures 3, 4), indicating a close relationship among them from different species (Ji et al., 2019; Yao et al., 2020). The biological function of *CsHSP20* genes can be predicted based on the function of similar genes in other species. Several subgroups in this study, including CIII, CX, CXI, CIX, and CVII, were not identified from the CsHSP20 genes of cucumber. The same happened in the other species, such as in rice where the HSP20 CVII and CIV subgroups were missing and in pepper where the HSP20 CIX, CV, CIV, CVIII, CXI, and CX subgroups were absent (Sarkar et al., 2009; Guo et al., 2015). This result indicates that gene loss and acquisition events are common in plant species. During evolution, the loss of *HSP20* gene may lead to the deletion of subpopulations.

Recent analysis results indicate that the average global temperature of the Earth is expected to increase by 1.5°C in the next two decades (Masson-Delmotte et al., 2021). Research shows that every 1°C increase in the annual average temperature will reduce the production of crops, such as rice, wheat, and corn by 3–8%. Therefore, studying the response mechanism of plants to high-temperature stress and improving the heat tolerance of crops are crucial in achieving world food security and human development. The Food and Agriculture Organization Corporate Statistical Database (FAOSTAT) shows that cucumber is widely cultivated in the world and its cultivated area is next only to that of tomato and onion (FAOSTAT, 2022). HSP20 plays a crucial role in extreme temperatures (Peffer et al., 2019; Wu et al., 2019; Cui et al., 2021; Li et al., 2021; Sun et al., 2022; Zhou et al., 2022). Therefore, it is very necessary to study the roles of *CsHSP20* genes under high temperature. According to the qRT-PCR conducted in this study, most of the *CsHSP20* genes responded rapidly to heat stress (Figure 7). This rapid response might be due to the compact gene structures with few introns (Figure 4). Studies have shown that a highly compact gene structure is conducive to achieve a fast and timely response from plants to diverse stresses (Ren et al., 2006). Moreover, genes with few or no introns were believed to have a high expression level in plants (Chung et al., 2006; Jeffares et al., 2008). These standpoints could be proven in other research. Although most of the *CsHSP20* genes in the current study showed a good response to heat stress, only two HSEs were present in their promoters, whereas many other elements were observed. Furthermore, a large proportion of the identified elements belonged to hormone and development related elements (Figure 6 and Supplementary Table 5), indicating that CsHSP20s are involved in multiple or specific functions of growth and development. As we know, plant hormones are signal compounds that regulate the key aspects of plant growth, development, and environmental stress response. Among the nine common plant hormones in plants, five of them, including ABA, auxin, BR, GA, and MeJA, play significant roles in response to heat stress (Waadt et al., 2022). In response to heat stress, BES1, a key transcription factor in the BR signaling pathway, is activated and directly binds to HSEs, known binding sites of HSFs, to induce the expression of HSPs (Albertos et al., 2022). The sHSP22 heat shock protein can be induced by auxin (Li et al., 2018). These conditions mean that the expression of heat shock proteins is closely related to hormones. Thus, even though there are no heat-responsive *cis*-elements in the promoter of the CsHSP20s gene, their expression can still be induced. With regard to how *CsHSP20* genes participate in the response to high temperatures, further research is needed. Notably, the relative expression of the *CsHSP20* genes (*CsHSP36.1*, *17.8*, *25.5*, *16.3*, *18.2*, *17.3*, *23.4*, *22.2A*, *18.1*, *23.8A*, *23.8B*, and *15.9*) increased hundreds or even thousands of times after 3 h of heat stress. The 12 *CsHSP20* genes may have been primarily involved in the biological pathway of heat stress and could be used as candidate genes for cucumber heat tolerance breeding. CsHSP20s not only contributed to the formation of biological heat tolerance, but also played important roles in the process of plant adaptation to the environment.

## Conclusion

In this study, we identified 30 *CsHSP20* genes in cucumber and characterized their chromosomal location, physicochemical properties, synteny, homologous gene pairs, phylogenetic tree, gene structure, conserved domains, PPI network, and *cis*-elements. The expression of the *CsHSP20* genes under high temperatures was investigated through qRT-PCR, and the results showed that the majority of *CsHSP20* genes responded to the heat stress treatment. The rapid upregulation of *CsHSP20* genes expression at high temperatures indicates that *CsHSP20* genes play vital roles in the acquired thermotolerance of cucumber. This study can provide a comprehensive foundation for the *CsHSP20* gene family and can be helpful in screening candidate genes for breeding new cucumber varieties with heat stress tolerance.

## Data availability statement

The datasets presented in this study can be found in online repositories. The names of the repository/repositories and accession number(s) can be found in the article/Supplementary material.

## Author contributions

JH, WL, and HW designed the experiments and wrote the manuscript. ZH, RW, YY, and XC performed the experiments and contributed to data analysis. All authors read and approved the manuscript.

## Footnotes

1
http://www.cucurbitgenomics.org/

2
http://pfam.sanger.ac.uk

3
http://smart.embl-heidelberg.de/

4
https://web.expasy.org/protparam/

5
http://linux1.softberry.com/

6
http://gsds.gao-lab.org/index.php

7
http://meme-suite.org/tools/meme

8
http://bioinformatics.psb.ugent.be/webtools/plantcare/html/

9
https://cn.string-db.org/
